# Competition between Li^+^ and Na^+^ in sodium transporters and receptors: Which Na^+^-Binding sites are “therapeutic” Li^+^ targets?†
†Electronic supplementary information (ESI) available: Table S1 listing all PDB entries of transporter and receptor structures with Na^+^ bound in the allosteric sites. Table S2 listing the relative % SASA and hydrogen bonds for the Na^+^-coordinating residues in the representative PDB structures. See DOI: 10.1039/c7sc05284g


**DOI:** 10.1039/c7sc05284g

**Published:** 2018-04-02

**Authors:** Todor Dudev, Karine Mazmanian, Carmay Lim

**Affiliations:** a Faculty of Chemistry and Pharmacy , Sofia University , Sofia 1164 , Bulgaria . Email: todorminkov2013@gmail.com; b Institute of Biomedical Sciences , Academia Sinica , Taipei 11529 , Taiwan . Email: carmay@gate.sinica.edu.tw; c Chemical Biology and Molecular Biophysics Program , Taiwan International Graduate Program , Academia Sinica , Taipei 11529 , Taiwan; d Department of Chemistry , National Tsing Hua University , Hsinchu 300 , Taiwan

## Abstract

Sodium (Na^+^) acts as an indispensable allosteric regulator of the activities of biologically important neurotransmitter transporters and G-protein coupled receptors (GPCRs), which comprise well-known drug targets for psychiatric disorders and addictive behavior. How selective these allosteric Na^+^-binding sites are for the cognate cation over abiogenic Li^+^, a first-line drug to treat bipolar disorder, is unclear. Here, we reveal how properties of the host protein and its binding cavity affect the outcome of the competition between Li^+^ and Na^+^ for allosteric binding sites in sodium transporters and receptors. We show that rigid Na^+^-sites that are crowded with multiple protein ligands are well-protected against Li^+^ attack, but their flexible counterparts or buried Na^+^-sites containing only one or two protein ligands are vulnerable to Li^+^ substitution. These findings suggest a novel possible mode of Li^+^ therapeutic action: By displacing Na^+^ bound by ≤2 protein ligands in buried GPCR sites and stabilizing the receptor's inactive state, Li^+^ could prohibit conformational changes to an active state, leading to lower cytosolic levels of activated guanine nucleotide-binding proteins, which are hyperactive/overexpressed in bipolar disorder patients.

## Abstract 2

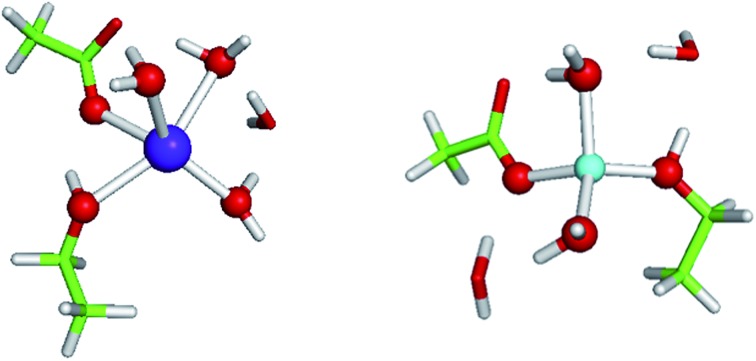
Li^+^ (turquoise), the better charge acceptor, can displace Na^+^ (purple) bound by only one or two aa residues in buried sites. Thus, Li^+^ can displace Na^+^ bound by Asp^–^ and Ser in the A_2A_AR/β_1_AR receptor and enhance the metal site's stability, thus prohibiting structural distortions induced by agonist binding, leading to lower cytosolic levels of activated G-proteins, which are hyperactive in bipolar disorder patients.

## Introduction

Lithium (Li^+^), a cation with unknown biological function in mammals, has been used (in the form of soluble salts) as a first-line mood stabilizer for people with depressive conditions, particularly those suffering from bipolar disorder.^1^,^2^ It also has beneficial effects in ameliorating damages induced by traumatic brain injury,^3^ demyelinating diseases,^4^,^5^ and chronic neurodegenerative diseases such as Alzheimer's, Parkinson's, and Huntington's diseases.^1^,^6^ Although the clinical and pharmacodynamical/pharmacokinetic effects of Li^+^ therapy have been known for decades, its mechanism of therapeutic action remains unclear. Several hypotheses have been put forth, most of which focus on the competition or cooperation between Li^+^ and native ions such as Mg^2+^ and Na^+^ in biological compartments.

One of the leading hypotheses posits that Li^+^ competes with Mg^2+^ in signal-transducing metalloenzymes that are overexpressed in bipolar patients such as glycogen synthase kinase 3β, inositol monophosphatase, and inositol polyphosphate phosphatase.^2^,^7^–^10^ By displacing weakly-bound Mg^2+^, Li^+^ inhibits these enzymes and eventually normalizes cell signaling. Subsequent calculations^9^ delineated the key factors controlling the outcome of the competition between Mg^2+^ and free, hydrated Li^+^ in proteins and elucidated why Li^+^ can replace Mg^2+^ only in enzymes known to be targets of Li^+^ therapy, but not in Mg^2+^-enzymes essential for cell function: Li^+^ can replace Mg^2+^ bound to only a few bulky ligands in sites with a net high positive charge that is reduced upon Li^+^ substitution. These findings were subsequently confirmed by ^7^Li magic-angle-spinning solid-state NMR experiments on *E. coli SuhB* inositol monophosphatase.^11^

Instead of competing with Mg^2+^, Li^+^ can co-bind with the native dication to modulate the properties of metal-loaded nucleotides such as adenosine triphosphate (ATP) and guanosine triphosphate (GTP), which act as activators of signal-transducing proteins.^12^–^14^ The bimetallic nucleotide complex can modulate the response of the host receptor and thus, the downstream signal transduction cascade in the cell: the binuclear ATP–Mg–Li complex, when bound to the neuronal P2X receptor (a ligand-gated Ca^2+^ channel), has been shown to elicit longer P2X activation than the native ATP–Mg cofactor.^12^ Subsequent calculations revealed that Li^+^ binding to [ATP–Mg]^2–^ does not alter the native cofactor's overall charge, conformation, and interactions with protein ligands lining the P2X binding site.^13^,^14^ However, if the protein matrix prevented ADP from binding Li^+^ in its preferred solution mode, then Li^+^ binding to ATP–Mg could reduce the native cofactor's susceptibility to hydrolysis, resulting in prolonged residence of the bimetallic ATP–Mg–Li complex in P2X and thus, prolonged receptor response.^14^ On the other hand, compared to the native GTP–Mg cofactor, bimetallic GTP–Mg–Li interacts less well with guanine nucleotide-binding proteins (G-proteins), which are hyperactive/overexpressed in bipolar disorder patients. Thus, Li^+^ binding to [GTP–Mg]^2–^ results in lower cytosolic levels of activated G-proteins and eventually normalizes cell signaling.^14^

Another hypothesis posits that Li^+^, in its free hydrated state, can compete with Na^+^. Bipolar disorder patients have abnormally high Na^+^ cytosolic concentrations.^15^,^16^ Monovalent Li^+^, by entering the cytosol *via* sodium channels, accumulates inside the cell lowering the intracellular concentration of Na^+^, which in turn reduces the Ca^2+^ cytosolic level. Decreasing the intracellular concentrations of both Na^+^ and Ca^2+^ attenuates the cell excitability, eventually normalizing the neuronal activity in bipolar disorder patients.^15^

However, another possible mode of Li^+^ therapeutic action has not been considered (to our knowledge); *viz.*, the competition between Li^+^ and Na^+^ for specific Na^+^-binding sites in signal-transducing proteins, notably neurotransmitter transporters and G-protein coupled receptors (GPCRs), which comprise well-known drug targets for psychiatric disorders^17^,^18^ and addictive behavior.^19^ Many transport proteins involved in trafficking of neurotransmitters such as glycine, serotonin, dopamine, glutamate, and γ-aminobutyric acid (GABA) across the cell membrane bind Na^+^, which acts as an indispensable allosteric regulator of their activities.^20^–^22^ Membrane receptors such as glutamate receptors and GPCRs that mediate excitatory transmissions are also subject to Na^+^ allosteric control.^23^,^24^ Several fundamental questions regarding the competition between Li^+^ and Na^+^ in these systems have not been addressed:

(1) What are the key determinants controlling the selectivity for Na^+^ over Li^+^ in Na^+^-proteins?

(2) Which Na^+^-binding sites are more vulnerable to Li^+^ substitution? If Li^+^ can displace Na^+^ from its binding site, how would this affect the host protein function?

Here, we aim to answer these questions. First, we surveyed the Protein Data Bank (PDB)^25^ for neurotransmitter transporters and GPCRs containing functional Na^+^-binding sites to determine their characteristic features. Then, we evaluated the thermodynamic outcome of the competition between Na^+^ and Li^+^ in various model transporter/receptor Na^+^-binding sites, as described in Methods. We reveal how this outcome depends on properties of the host protein and its binding cavity and which Na^+^-sites are prone to Li^+^ substitution. Our findings suggest a novel possible mode of Li^+^ therapeutic action.

## Methods

### PDB survey

The PDB^41^ was searched for structures of neurotransmitter transporters and GPCRs that contain bound Na^+^; the resulting PDB entries are listed in ESI Table S1.† Na^+^-bound ligands were identified by a cutoff of 3 Å. If the same protein has more than one structure, the highest-resolution one with the most number of Na^+^-bound ligands was chosen. This yielded 16 representative proteins (Table 1) with distinct Na^+^-sites. For a given structure, we computed the % ratio of the solvent-accessible surface area (SASA) of the residue X in the protein to the accessible surface area of X in the tripeptide –Gly–X–Gly– using the MOLMOL^42^ program with a solvent probe radius of 1.4 Å. For a given structure, we also computed all hydrogen bonds made by the Na^+^-ligating residues using the HBPLUS Hydrogen Bond Calculator version 3.15.^43^ The relative % SASA and hydrogen bonds made by the Na^+^-ligating residues in the representative PDB structures are listed in ESI Table S2.†

**Table 1:** Representative sodium transporters and receptors containing Na^+^-binding sites

PDB entry	Resol^*n*^ (Å)^*a*^	Transporter proteins	Na^+^-bound ligands^*b*^
2A65 26	1.65	Leucine transporter LeuT	Na1: A22^bb^ N27 T254(O^G1^, O) N286 Leu (substrate)
			Na2: G20^bb^ V23^bb^ A351^bb^ T354 S355
3KBC 27	3.51	Glutamate transporter GltPh	Na1: G306^bb^ N310^bb^ N401^bb^ D405(O^D1^; O^D2^)
			Na2: T308 S349^bb^ I350^bb^ T352^bb^
4XP1 28	2.89	Dopamine transporter DAT	Na1: A44^bb^ N49 S320^bb^ S320 N352 H_2_O
			Na2: G42^bb^ V45^bb^ L417^bb^ D420 S421
5E9S 29	2.80	Glutamate transporter GltTk	Na1: G309^bb^ N313^bb^ N405^bb^ D409(O^D1^ O^D2^)
			Na2: T311^bb^ S352^bb^ I353^bb^ T355^bb^
			Na3: Y91^bb^ T94 S95 N313 D315
5I71 30	3.15	Serotonin transporter SERT	Na1: A96^bb^ N101 S336^bb^ S336 N368^bb^
			Na2: G94^bb^ V97^bb^ L434^bb^ D437 S438 H_2_O
5LM4 31	3.10	Glutamate transporter GLAST1/EAAT1	T376^bb^ S417^bb^ I418^bb^ A420^bb^

**Receptor proteins**			
3C32 23	1.72	Glutamate receptor kainate GluK1 (GluR5)	E96(O^E1^; O) I99^bb^ D100 H_2_O
3G3F 32	1.38	Glutamate receptor kainate GluK2 (GluR6)	E97(O^E1^; O) I100^bb^ D101 H_2_O
3OM1 33	1.68	Glutamate receptor kainate GluK5 (KA2)	F162^bb^ S165^bb^ E167^bb^ 2(H_2_O)
3S9E 34 (chain B)	1.60	Glutamate receptor kainate GluK3 (GluR7)	Na1: S24^bb^ R26^bb^ 4(H_2_O)
			Na2: N5^bb^ S52 2(H_2_O)
3VW7 35	2.20	GPCR receptor protease-activated PAR1	D148 S189 D367 2(H_2_O)
4BVN 36	2.10	GPCR receptor adrenergic β_1_AR	Na1: C192^bb^ D195^bb^ C198^bb^ 2(H_2_O)
			Na2: D87 S128 3(H_2_O)
4EIY 37	1.80	GPCR receptor adenosine A_2A_AR	D52 S91 3(H_2_O)
4LDE 38	2.79	GPCR receptor adrenergic β_2_AR	N1103 C1184^bb^ E1187^bb^ C1190^bb^ H_2_O
4N6H 39	1.80	GPCR receptor opioid δ-OR	D95 N131 S135 2(H_2_O)
5NDD 40	2.80	GPCR receptor protease-activated PAR2	D121 D340 N158 S162 N33

^*a*^Resolution of the PDB structure.

^*b*^Residues with a superscript “bb” bind to Na^+^*via* their backbone carbonyl groups; otherwise, they bind Na^+^*via* their sidechain O atoms. Atoms in parenthesis after a residue denotes that both atoms binds to Na^+^.

### Models used

On the basis of the structure/composition of Na^+^-sites from the protein crystal structures, the Na^+^-binding sites (without the substrate) were modeled as mononuclear penta/hexacoordinated complexes, comprising mostly neutral O-containing protein ligands (see Table 1 and Results). *N*-Methylacetamide (CH_3_CONHCH_3_) was used to model the backbone peptide group as well as the Asn/Gln side chain rather than acetamide (CH_3_CONH_2_), as replacing a H from the acetamide NH_2_ with a methyl group does not alter metal binding and *N*-methylacetamide and acetamide have similar solvation free energies.^44^,^45^ The Ser/Thr and Asp^–^/Glu^–^ side chains were modeled as ethanol (CH_3_CH_2_OH) and acetate (CH_3_COO^–^), respectively.

### Geometry optimization

Among various combinations of *ab initio* methods/density functionals (HF, MP2, SVWN, B3LYP) and basis sets (6-31+G(d,p), 6-31+G(2d,2p), 6-31+G(3d,p), 6-31+G(3d,2p), 6-311++G(d,p) and 6-311++G(3df,3pd)), the B3-LYP/6-31+G(3d,p) method has been found to be the most efficient in reproducing experimental observables: it could reproduce (to within experimental error) the dipole moments of water and protein ligands,^46^ bond distances between oxygen and various cations (Na^+^, K^+^, Li^+^, Mg^2+^, Ca^2+^, Fe^3+^, and Ga^3+^) in aqua and crown ether complexes^9^,^46^–^49^ and the free energies of metal (Li^+^, Na^+^, K^+^, Mg^2+^ and Ca^2+^) exchange in crown ether, acetate, oxalate and nitrilotriacetic acid complexes.^9^,^46^,^50^ Hence, it was used to optimize the geometry of each metal complex and to compute the electronic energies, *E*_el_, using the Gaussian 09 program.^51^

Initially, we built hexacoordinated complexes with all six protein and/or water ligands bound to Na^+^. During geometry optimization, some hexacoordinated Na^+^ complexes isomerized to more stable pentacoordinated structures with a ligand relegated to the second shell. These were retained for further evaluation. The fully optimized Na^+^ complexes were used as starting structures for optimizing the respective Li^+^ counterparts. In the fully optimized Li^+^ complexes, two ligands spontaneously moved to the metal's second shell, leaving four ligands coordinated to Li^+^, consistent with lithium's known preference for tetrahedral complexes.^52^,^53^ Frequency calculations for each optimized construct were performed at the same level of theory – no imaginary frequency was found in any of the fully optimized structures.

### Reaction modeled

Based on the fully optimized structures, we computed the free energy Δ*G*^*ε*^ for eqn (1)

1 [Li^+^-aq] + [Na^+^-protein] → [Li^+^-protein] + [Na^+^-aq]

where [M^+^-protein] and [M^+^-aq] (M^+^ = Li^+^ or Na^+^) represent the cation bound to protein ligands in the binding cavity and unbound in its vicinity, respectively. The cation exchange Δ*G*^*ε*^ in a binding cavity characterized by an effective dielectric constant *ε* can be evaluated as

2 Δ*G*^*ε*^ = Δ*G*^1^ + Δ*G*_solv_^*ε*^([Li^+^-protein]) + Δ*G*_solv_^*ε*^(Na^+^) – Δ*G*_solv_^*ε*^([Na^+^-protein]) – Δ*G*_solv_^*ε*^(Li^+^)

where Δ*G*^1^ is the gas-phase free energy for eqn (1) and Δ*G*_solv_^*ε*^ is the solvation free energy Δ*G*_solv_^*ε*^. To mimic varying solvent exposure of the metal-binding cavity, *ε* was varied from 4 to 30.

### Computing the gas-phase free energy, Δ*G*^1^

This was computed from the differences in the electronic energies (Δ*E*_el_), thermal energies (Δ*E*_th_) and entropies (Δ*S*) between the products and reactants in eqn (1) at a temperature *T* of 298.15 K according to:

3 Δ*G*^1^ = Δ*E*_el_ + Δ*E*_th_ – *T*Δ*S*

In evaluating the thermal energies and entropies, the vibrational frequencies were scaled by an empirical factor of 0.9613.^54^ The basis set superposition error and dispersion correction for eqn (1) were found to be insignificant (<1.5 kcal mol^–1^),^46^ thus they were not included.

### Computing the solvation free energy, Δ*G*_solv_^*ε*^

This was computed by solving Poisson's equation with natural bond orbital atomic charges^55^ using the MEAD (Macroscopic Electrostatics with Atomic Detail) program.^56^ The effective solute radii were obtained by adjusting the CHARMM^57^ van der Waals radii to reproduce the experimental hydration free energies of Li^+^, Na^+^, and model ligands to within 1.1 kcal mol^–1^.^9^,^58^ The resulting values are (in Å): *R*_Li_ = 1.38, *R*_Na_ = 1.70, *R*_C_ = 1.95, *R*_N_ = 1.75, *R*_O_(–CONH–) = 1.72, *R*_O_(–CH_2_OH) = 1.90, *R*_O_(H_2_O/Na–H_2_O) = 1.85, *R*_O_(Li–H_2_O) = 1.84, *R*_O_(COO) = 1.40, *R*_H_ = 1.50, *R*_H_(Na–H_2_O) = 1.26, and *R*_H_(Li–H_2_O) = 1.44.

## Results

### Analysis of PDB structures

The results in Table 1 show some interesting differences between sodium transporters and receptors in terms of the number and composition of allosteric Na^+^-binding sites: sodium transporters generally possess two or more relatively “dry” juxtaposed Na^+^ sites where each Na^+^ is bound by mostly protein ligands rather than water molecules. In contrast, sodium receptors exhibit only one Na^+^-binding site, where the Na^+^ is generally bound by fewer (≤3) protein ligands and up to four water molecules.

We further analyzed the sodium transporters and receptors in Table 1 for the number and type of ligands bound to Na^+^ as well as the relative burial and flexibility of allosteric Na^+^-binding sites. A Na^+^-binding site is deemed to be buried if the average relative SASA of the Na^+^-ligating amino acid (aa) residues is <20%, partially buried if it is between 20 and 50%, and solvent accessible if it is >50% (see ESI Table S2†).^59^ A Na^+^-binding site with a dense hydrogen-bonding network that would presumably prohibit structural reorganization of Na^+^-site maybe considered to be relatively rigid. The results in Table 2 show the following features common to allosteric Na^+^-binding sites in both sodium transporters and receptors:

**Table 2:** Composition and the relative burial/rigidity of Na^+^-binding sites in representative sodium transporters and receptors

Na^+^ CN^*a*^	# of H_2_O ligands^*b*^	# of neutral aa ligands^*c*^	# of anionic ligands^*d*^	PDB entries^*e*^	Buried^*f*^	Total # of HBs^*g*^
4	—	1 OH + 3 amides	0	*3KBC (Na2)*	Yes	8
4	—	4 amides	0	*5LM4*	Yes	4
4	—	4 amides	0	5E9S (Na2)	Yes	8
5	2	3 amides	0	**4BVN (Na1)**	Partially (23%)	7
5	2	3 amides	0	**3OM1**	Yes	4
5	—	2 OH + 3 amides	0	2A65 (Na2)	Yes	10
6	—	1 OH + 5 amides	0	2A65 (Na1)	Yes	12
6	1	1 OH + 4 amides	0	4XP1 (Na1)	Yes	8
5	—	1 OH + 4 amides	0	*5I71 (Na1)*	Yes	11
5	3	1 OH	1	**4EIY**	Yes	5
				**4BVN (Na2)**	Yes	9
5	2	1 OH + 1 amide	1	**4N6H**	Yes	15
5	—	3 amides	1 (Bi)	*3KBC(Na1)*	Yes	9
				5E9S (Na1)	Yes	12
6	1	1 OH + 3 amides	1	*5I71(Na2)*	Yes	10
5	—	1 OH + 3 amides	1	4XP1(Na2)	Yes	10
5	—	2 OH + 2 amides	1	5E9S (Na3)	Yes	16
5	—	1 OH + 2 amides	2	**5NDD**	Yes	14
5	1	2 amides	2	**3G3F**	Yes	11
				**3C32**	Yes	10
6	2	1 OH	2	**3VW7**	Yes	11
5	1	4 amides	—	**4LDE**	Yes	6
6	4	2 amides	—	**3S9E (Na1)**	Partially (40%)	3
4	2	1 OH + 1 amide	—	**3S9E (Na2)**	No (56%)	4

^*a*^The number of aa and water atoms bound to Na^+^.

^*b*^Number of Na^+^-bound water molecules.

^*c*^Number of Na^+^-bound neutral aa ligands. OH denotes Ser/Thr side chain and “amide” denotes backbone/Asn/Gln amide group.

^*d*^Number of Na^+^-bound negatively charged Asp^–^/Glu^–^ ligands. “Bi” means that both carboxylate oxygen atoms coordinate Na^+^.

^*e*^PDB entries in italics have resolution ≥3.0 Å; those in bold correspond to receptor proteins.

^*f*^The mean % SASA of all Na^+^-binding aa residues is given if it exceeds 20%.^59^

^*g*^The total number of hydrogen bonds formed by all the Na^+^-ligating aa residues (excluding Na^+^-bound water molecules).

(a) Na^+^-binding sites are mononuclear rather than bi/polynuclear with an average Na^+^···Na^+^ distance of 7.9 ± 1.1 Å in transporters containing multiple Na^+^-binding sites.

(b) Na^+^ is generally penta/hexacoordinated in receptors and transporters, albeit for structures with resolution >2.5 Å, the Na^+^ coordination number is uncertain since bound water molecule(s) may not be seen in the structure.

(c) Na^+^, being a “hard” cation, binds to “hard” oxygen atoms from the protein ligands.

(d) Na^+^ is bound by at least two aa ligands and no more than two negatively charged ones.

(e) Na^+^ is preferentially bound by neutral protein ligands (backbone carbonyl or small Ser/Thr/Asn side chains) rather than negatively charged ones (Asp^–^/Glu^–^), which generally bind Na^+^*via* one rather than both carboxylate oxygen atoms.

(f) Na^+^-binding sites in both transporter and receptor proteins are generally quite buried with multiple (≥3) hydrogen bonds (see also ESI Table S2†).

### Assessing the outcome of the Li^+^*vs.* Na^+^ competition in Na^+^-binding sites

**Fig. 1:**
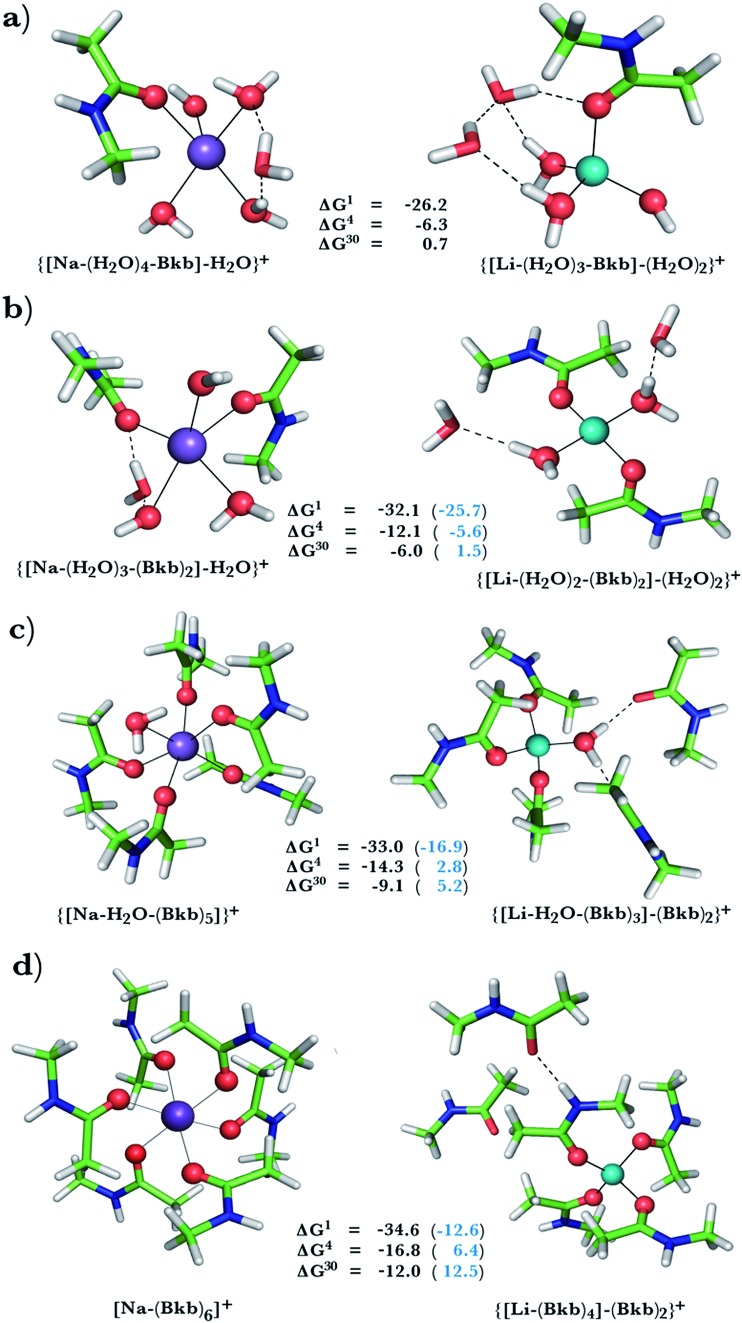
Calculated free energies and optimized structures of Na^+^ (purple) and Li^+^ (turquoise) complexes with amides. Metal-coordinating atoms are rendered as red spheres with non-coordinating ones in stick format with oxygen in red, carbon in green, nitrogen in blue, and hydrogen in white. Hydrogen bonds between first-shell and second-shell ligands are indicated by dash lines.

**Fig. 2:**
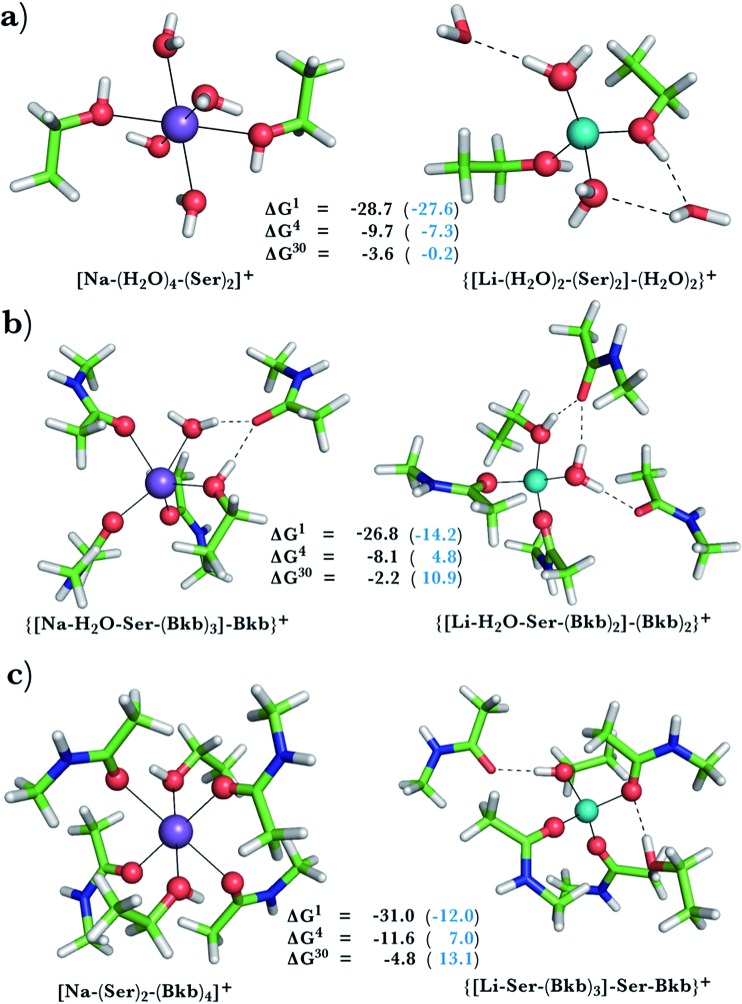
Calculated free energies and optimized structures of Na^+^ and Li^+^ complexes containing amide and hydroxyl ligands. Atom colors as in Fig. 1.

**Fig. 3:**
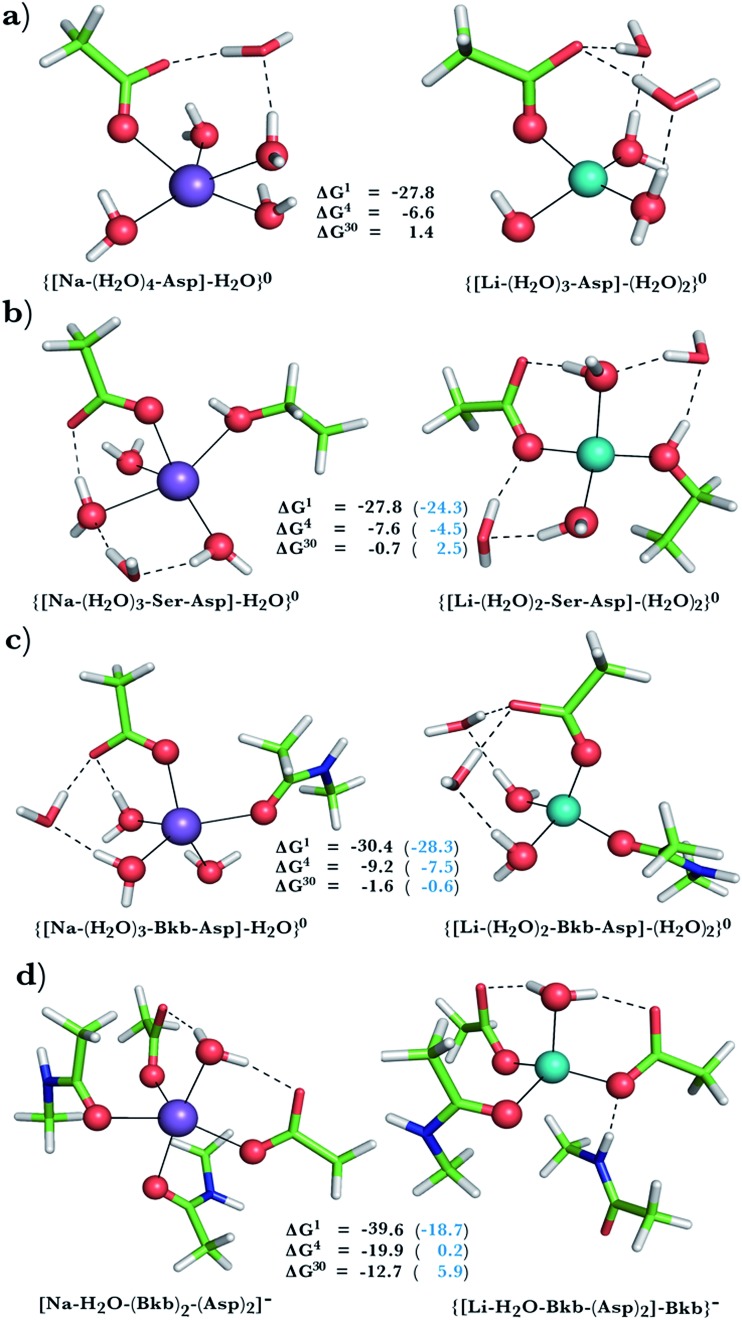
Calculated free energies and optimized structures of Na^+^ and Li^+^ complexes containing amide, hydroxyl and acidic ligands. Atom colors as in Fig. 1.

This was assessed by the free energies for replacing Na^+^ with Li^+^ in relatively buried (*ε* = 4) or partially solvent-exposed (*ε* = 30) protein binding pockets based on the fully optimized structures of Na^+^ and Li^+^ complexes shown in Fig. 1–3. As the protein matrix and the multiple hydrogen bonds formed by the Na^+^-ligating aa ligands may prohibit the incoming Li^+^ from adopting its favored tetrahedral ligand arrangement, we computed two sets of Na^+^ → Li^+^ free energies: those in black characterize cation competition in *flexible* Na^+^-sites that permit structural changes upon Li^+^ binding and those in blue portray the Na^+^ → Li^+^ exchange in *rigid* Na^+^-sites that prohibit structural reorganization. To model such inflexible binding sites, we fixed the binding cavity's geometry by freezing the protein ligand positions in the fully optimized Na^+^ complexes and replaced Na^+^ with Li^+^, followed by partial optimization, allowing Li^+^ and the water ligands to find their optimal positions.

A positive Δ*G*^*ε*^ in Fig. 1–3 implies a Na^+^-selective site, whereas a negative value implies a Li^+^-selective one. Below, we focus on how the Na^+^ → Li^+^ free energy changes with varying solvent exposure, rigidity, composition, and net charge of the metal-binding site rather than the absolute free energy. Note that the trends in the free energies computed using the approach herein have been found to be in accord with experimental observations in previous studies.^9^,^47^,^49^,^50^,^58^,^60^,^61^

### Na^+^ → Li^+^ exchange in complexes containing neutral ligands

Since Na^+^ is found coordinated to only *neutral* ligands in several proteins (Table 2), we optimized complexes of Na^+^ bound to neutral ligands such as the backbone peptide group (Bkb)/Asn side chain (modeled by *N*-methylacetamide) or Ser/Thr side chain (modeled by ethanol) and water molecules. *Flexible* Na^+^-binding sites containing many neutral amide residues are prone to Li^+^ substitution, as evidenced by the favorable Na^+^ → Li^+^ free energies in Fig. 1b–d. This is mainly because Li^+^ is a better charge acceptor and forms more favorable interactions with the ligands than the native Na^+^. Consequently, increasing the number of amides lining the flexible binding pocket enhanced the selectivity for Li^+^ over Na^+^: as the number of amide ligands increased from one (Fig. 1a) to two (Fig. 1b) to five (Fig. 1c) to six (Fig. 1d), the Δ*G*^*ε*^ (*ε* = 1–30) became more negative. Such electronic effects favoring Li^+^ over Na^+^ are attenuated in solvent-exposed sites, where lithium's higher desolvation penalty outweighs sodium's solvation free energy gain upon Na^+^ → Li^+^ substitution (Δ*G*^30^ less negative than Δ*G*^4^). Hence, buried Na^+^-sites seem to be more susceptible to Li^+^ attack than solvent-accessible ones.

However, if the Na^+^-binding sites containing many neutral amide residues were *rigid*, they would be well protected against Li^+^ attack (Fig. 1c and d, positive Δ*G*^4^/Δ*G*^30^ in blue). In contrast to flexible sites, the more crowded a rigid binding site, the more Na^+^-selective it becomes: as the number of amide ligands in a rigid binding site increased from two to six (Fig. 1b–d), the Na^+^ → Li^+^ free energy also increased; *e.g.*, Δ*G*^4^ (in blue) increased from –5.6 to 2.8 and 6.4 kcal mol^–1^, respectively. This is because in a rigid site, Li^+^ is forced to adopt sodium's longer bond distances, resulting in reduced charge transfer, as compared to flexible sites. Furthermore, the backbone/Asn amides often form hydrogen bonds in the Na^+^-transporter and receptor structures (see ESI Table S2†). Hence, increasing the number of amide ligands lining a rigid binding site might make it harder to restructure the protein cavity in favor of Li^+^: this is supported by an increase in the free energy penalty for restructuring the Na^+^-binding site upon Li^+^ substitution: the Δ*G*^4^ difference between rigid and flexible sites increased from 6.5 to 17 to 23 kcal mol^–1^ in going from Fig. 1b to d. Thus, rigid, crowded Na^+^-binding sites, in contrast to their flexible counterparts, prefer Na^+^ over Li^+^.

When we replaced one or two amide ligands in Fig. 1b–d by ethanol (modeling the Ser/Thr side chain), the above trends do not change. Thus, Na^+^-binding sites are selective for the cognate cation over non-native Li^+^ if they are *rigid* and comprise >2 neutral protein ligands (Fig. 1c/1d and 2b/2c, positive Δ*G*^4^/Δ*G*^30^ in blue). They become prone to Li^+^ substitution if they can undergo structural changes to meet lithium's coordination preferences (Fig. 2, negative Δ*G*^4^/Δ*G*^30^ in black). Compared to an amide however, the hydroxyl group is a poorer charge donor and its interactions with Li^+^ are attenuated more than those with Na^+^, making the Na^+^-site less susceptible to Li^+^ attack when one or two amide ligands are replaced by hydroxyl groups (less negative Δ*G*^4^/Δ*G*^30^ in Fig. 2a–c than those in Fig. 1b–d, respectively).

### Na^+^ → Li^+^ exchange in complexes comprising neutral and negatively charged ligands

Since Na^+^ is found bound to one or at most two acidic ligands in some proteins in Table 2, we also assessed the Na^+^*vs.* Li^+^ competition in complexes comprising one or two acetates (modeling Asp^–^/Glu^–^ carboxylates) and a few neutral ligands. As found above, increasing the number of protein ligands lining a *flexible* binding pocket favored the cation that is the stronger electron acceptor; *i.e.*, Li^+^ over Na^+^: as the number of protein ligands increased from one (Fig. 3a) to two (Fig. 3b/3c) to four (Fig. 3d), the Δ*G*^4^/Δ*G*^30^ became more negative. However, rigidifying the binding site, which forces the incoming Li^+^ to adopt unfavorable coordination distances rendered the most crowded binding pocket in the series Na^+^-selective (Fig. 3d, positive “blue” Δ*G*^*ε*^, *ε* > 4). As found above, substitution of an amide in Fig. 3c with OH (a weaker charge donor) in Fig. 3b yielded less negative Δ*G*^4^/Δ*G*^30^.

## Discussion

### Factors governing the competition between Li^+^ and Na^+^ in Na^+^-binding sites

The results herein reveal how the properties of the host protein and its binding cavity affect the outcome of the competition between Na^+^ and Li^+^ in Na^+^-binding sites:

#### (1) Relative rigidity

Rigid Na^+^-sites that force Li^+^ to adopt sodium's longer bond distances, resulting in weaker Li^+^–O bonds, enhance the selectivity for Na^+^ over Li^+^, whereas flexible Na^+^-sites that allow Li^+^ to adopt its optimal coordination distance/geometry are prone to Li^+^ attack. Thus, tuning the relative flexibility of the binding site (*e.g.*, by changing the number/strength of hydrogen-bonding interactions) may modulate selectivity for Na^+^ or Li^+^. Free energy simulations of LeuT transporter also underscore the rigidity of the Na^+^-binding site, which is enforced by the local structural restraints arising from hydrogen-bonding networks, in determining selectivity for the Na2-site.^58^,^62^

#### (2) Number and type of protein ligands

Increasing the number of protein ligands enhances selectivity for Na^+^ over Li^+^ in *rigid* binding sites. Neutral protein ligands that are *weak* charge-donors such as the Ser/Thr hydroxyl side chain favor Na^+^ over Li^+^ in *rigid* sites, whereas anionic ligands that are strong charge-donors such as the Asp/Glu carboxylates favor the cation with higher charge-accepting ability; *i.e.*, Li^+^ over Na^+^.

#### (3) Relative solvent accessibility

Allowing water into the binding cavity favors the cation with smaller dehydration penalty; *i.e.*, Na^+^ over Li^+^, whereas a solvent-inaccessible cavity enhances metal–ligand interactions and favors Li^+^, which can accept more charge and form stronger ligand interactions than Na^+^.

### Comparison with experiment

Our findings are in line with available experimental data on sodium transporters/receptors. The calculations predict that rigid Na^+^-binding sites containing >3 protein ligands would be well-protected against Li^+^ attack (positive “blue” Δ*G*^4^/Δ*G*^30^ values for metal complexes comprising >3 neutral protein ligands in Fig. 1c, d, 2b and c). Our PDB analyses show that such rigid Na^+^-binding sites crowded with >3 protein ligands, which form hydrogen-bonding networks, are found in most of the sodium transporters and receptors listed in Table 1. In accord with our findings, the Na(1)-binding sites in neurotransmitter transporters have been experimentally found to be especially well-protected from Li^+^ substitution.^63^–^65^ Furthermore, experiments on aspartate^20^ and glutamate^66^ transporters using Li^+^ concentrations of 100 and 150 mM, respectively, show that Li^+^ indeed binds with lower affinity to these allosteric Na^+^-binding sites than the native metal co-factor.

**Fig. 4:**
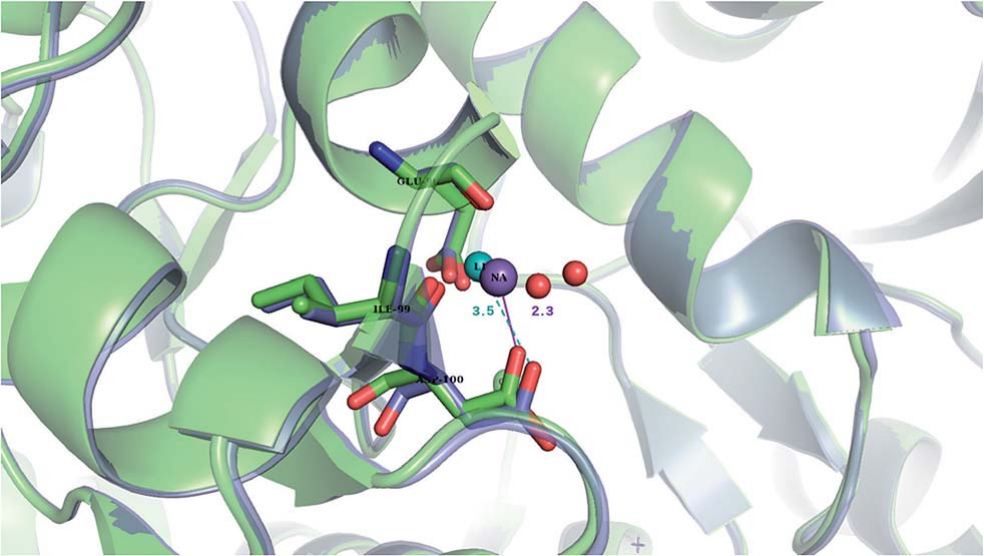
The superimposed X-ray structures of Na^+^ (; 3C32) and Li^+^ (; 3C31) binding sites in the glutamate kainate receptor (GluR5). The Li^+^ structure is in purple with Li^+^ in turquoise and the Na^+^ structure is in green with Na^+^ in purple. The respective metal-binding residues are depicted as sticks with nitrogen in blue and oxygen in red. Distances from Asp100 to the cation are indicated.

Interestingly, X-ray structures of the glutamate receptor 5 bound to Na^+^ (PDB entry ; 3C32) and to Li^+^ (PDB entry ; 3C31)^23^ have been solved to a resolution of 1.72 and 1.49 Å, respectively. The Na^+^-bound ; 3C32 structure shows Na^+^ bound to two neutral (Glu96 O, Ile99 O) and two acidic (Glu96 O^E1^, Asp100 O^D1^) aa ligands that altogether form ten hydrogen bonds (see Tables 1 and S2†). Superimposition of the Na^+^ and Li^+^-bound structures in Fig. 4 shows a rigid Na^+^-site that does not change its original structure/size upon accommodating Li^+^: the three protein ligands (Glu96, Asp100, and Ile99) that coordinate Na^+^ adopt similar positions/orientations, but Li^+^ is closer to Glu96 (Li^+^–O^OE1^ = 2.0 Å) at the expense of losing contact with Asp100 (Li^+^–O^OD1^ = 3.5 Å). Thus, Li^+^ does not fit favorably in the rigid cavity and binds with lower affinity than the cognate Na^+^ to the glutamate receptor 5.^23^ Consistent with this experimental finding, we find that in a rigid cavity, Li^+^ cannot successfully compete with Na^+^ bound to two amides and two carboxylates (Fig. 3d, positive “blue” Δ*G*^*ε*^, *ε* > 4).

### Biological implications: Na^+^-binding sites vulnerable to Li^+^ attack

The calculations reveal that *solvent-inaccessible* Na^+^-binding sites lined by only one or two aa residues are susceptible to Li^+^ attack, regardless of the aa type and relative rigidity of pocket (negative Δ*G*^4^ in Fig. 1a, b, 2a, and 3a–c). Notably, they predict that Li^+^ can displace Na^+^ bound by Asp^–^ and Ser in a *buried* Na^+^-binding site (Fig. 3b, negative Δ*G*^*ε*^, *ε* < 30). Such a deeply buried Na^+^-binding site comprising an Asp^–^ and a Ser is found in two GPCRs; *viz.*, the A_2A_AR adenosine receptor (PDB entry ; 4EIY) and the β_1_AR adrenergic receptor (PDB entry ; 4BVN, Na2-site). Bound Na^+^ allosterically controls the GPCR activity: It stabilizes the inactive/resting receptor conformation, which cannot transmit signal to the G-protein and associated effectors.^24^,^37^,^67^ Agonist binding to the receptor disrupts the allosteric Na^+^-binding site (by activating transmembrane helix motions) and translocates Na^+^ to another protein locality,^24^ causing conformational changes that activate the receptor and trigger G-protein downstream signaling.

The aforesaid mechanism of allosteric regulation by Na^+^ suggests that factors stabilizing the allosteric Na^+^-binding site would prohibit structural distortions induced by agonist binding, leading to lower cytosolic levels of activated G-proteins, which are hyperactive/overexpressed in bipolar disorder patients. Here, we propose that such a factor is Li^+^, which can displace Na^+^ in a buried site (modeled in Fig. 3b). Molecular dynamics simulations (despite limitations of the classical force fields^68^,^69^) also suggest that Li^+^ coordination to such an allosteric binding site is energetically favored over Na^+^ binding.^70^ Compared with the cognate Na^+^ cation, Li^+^, the better charge acceptor, forms stronger interactions with Asp^–^ and Ser and enhances the stability of the allosteric binding pocket, leading to decreased G-protein activity in the cell. Such effects have indeed been observed in a recent study^71^ of the GluK2/K5 receptor heterodimer, which showed that Li^+^ could stabilize the heterodimer and slow its desensitization.

Lithium's replacement of Na^+^ can not only lock the GPCR protein in an inactive state, but also disrupt conformational transitions in neurotransmitter Na^+^ symporters, thus compromising substrate transport.^72^,^73^ Furthermore, it can alter the selectivity for the co-transported substrate, as found for the neuronal excitatory amino-acid transporter 3 (EAAT3), also known as EAAC1.^74^ Thus, lithium's replacement of Na^+^ in sodium transporter/receptor proteins could affect their biological functions. Conversely, Li^+^ might be scavenged by certain sodium proteins before it can reach its intracellular targets (see Introduction), hence it would be interesting to assess if Li^+^ could displace Na^+^ in Na-proteins that are ubiquitous in the blood in future studies.

## Conclusion

This is the first study delineating the key factors determining the outcome of the competition between Li^+^ and Na^+^ for allosteric Na^+^-binding sites in biologically important neurotransmitter transporters and GPCRs. We show that abiogenic Li^+^ can target those Na^+^-binding sites that are flexible with multiple neutral protein ligands or those that are buried containing only one or two aa ligands. By displacing Na^+^ bound to an Asp^–^ and a Ser in the solvent-inaccessible metal-binding sites of the A_2A_AR adenosine and the β_1_AR adrenergic GPCRs, Li^+^ could stabilize the receptor's inactive state by prohibiting conformational changes to the active state, thus leading to decreased G-protein activity, which are hyperactive in bipolar patients.

## Conflicts of interest

The authors declare no competing financial interest.

## Supplementary Material

Supplementary information
