# Supplementary material for: Competition between Li+ and Na+ in sodium transporters and receptors: Which Na+-Binding sites are “therapeutic” Li+ targets?
Source: Chem Sci. 2018 Apr 2;9(17):4093–103. doi: 10.1039/c7sc05284g (PMC5944251; doi:10.1039/c7sc05284g)
Supplement: Supplementary file 1 [file SC-009-C7SC05284G-s001.pdf]

Electronic Supplementary Material (ESI) for Chemical Science.
This journal is © The Royal Society of Chemistry 2018

Supporting Information

Competition Between Li^+ and Na^+ in Sodium Transporters and Receptors: Which Na^+-Binding Sites are "Therapeutic" Li^+ Targets?

Todor Dudev,[a,*] Karine Mazmanian,[b,c] and Carmay Lim [b,d,*]
[a]Faculty of Chemistry and Pharmacy, Sofia University, Sofia 1164, Bulgaria

[b]Institute of Biomedical Sciences, Academia Sinica, Taipei 11529, Taiwan

[c]Chemical Biology and Molecular Biophysics Program, Taiwan International Graduate Program, Academia Sinica, Taipei 11529, Taiwan

[d]Department of Chemistry, National Tsing Hua University, Hsinchu 300, Taiwan

Table S1. PDB entries of the transporter and receptor structures with Na^{+}.[a]

PDB ID	Protein	Resolution (Å)	CATH code	Other PDB structures with sodium
2A65	Leucine transporter LeuT	1.65	N/A	2Q6H, 2Q72, 2QB4, 2QEI, 3F3A, 3F3C, 3F3D, 3F3E, 3F48, 3F4I, 3F4J, 3GJC, 3GJD, 3GWU, 3GWV, 3GWW, 3MPN, 3MPQ, 3QS4, 3QS5, 3QS6, 3TT1, 3TT3, 3TU0, 3USG, 3USI, 3USJ, 3USK, 3USL, 3USM, 3USO, 3USP, 4FXZ, 4FY0, 4HMK, 4HOD, 4MM4, 5JAE, 5JAF, 5JAG
3KBC	Aspartate transporter GltPh	3.51	1.10.38 60.10	2NWX, 3KBC, 3V8F, 3V8G, 4IZM, 4OYF, 4X2S, 5CFY
4XP1	Dopamine transporter DAT	2.89	N/A	4M48, 4XNX, 4XP1, 4XP4, 4XP5, 4XP6, 4XP9, 4XPA, 4XPB, 4XPF, 4XPG, 4XPH, 4XPT
5E9S	Glutamate transporter GltTk	2.80	N/A	N/A
5I71	Serotonin transporter SERT	3.15	N/A	5I6X, 5I71, 5I73, 5I75, 6AWN, 6AWO, 6AWP, 6AWQ
5LM4	Glutamate transporter GLAST1/EAAT	3.10	N/A	5LLM, 5LLU, 5LM4
3C32	Glutamate receptor kainate GluK1 (GluR5)	1.72	3.40.19 0.10	N/A
3G3F	Glutamate receptor kainate GluK2 (GluR6)	1.38	3.40.19 0.10	2XXR, 2XXT, 2XXU, 3G3F, 3G3G, 3G3H, 3G3I, 3G3J, 3G3K, 3OM1, 4BDL, 4BDN, 4BDO, 4BDQ, 4BDR
3S9E	Glutamate receptor kainate GluK3 (GluR7)	1.60	3.40.19 0.10	N/A
3OM1	Glutamate receptor kainate GluK5 (KA2)	1.68	3.40.50 .2300	N/A
3VW7	GPCR receptor Protease-activated PAR1	2.20	1.20.10 70.10 1.10.53 0.40	N/A

4BVN	GPCR receptor Adrenergic b1AR	2.10	1.20.10 70.10	2VT4, 2Y00, 2Y02, 2Y03, 2Y04, 2YCW, 2YCY, 3ZPQ, 3ZPR, 4AMJ, 4BVN, 5A8E
4EIY	GPCR receptor Adenosine A2AAR	1.80	1.20.10 70.10 1.20.12 0.10	N/A
4LDE	GPCR receptor Adrenergic b2AR	2.79	1.10.53 0.40 1.20.10 70.10	4LDE, 4LDL, 4LDO, 4QKX
4N6H	GPCR receptor Opioid d-OR	1.80	1.20.12 0.10 1.20.10 70.10	4N6H, 4RWD
5NDD	GPCR receptor Protease-activated PAR2	2.80	N/A	5NDD, 5NDZ

Table S2. Relative % SASA and hydrogen bonds (HBs) made by the Na^+-coordinating residues in the representative PDB structures.

Na^+-binding residues	% SASA	# of HB (as an acceptor)	# of HB (as a donor)	HB-partner residues
**Leucine transporter LeuT 2A65**				
**Na1:**				
Ala22	0.41	—	1	$Met18^{MM}$
Asn27	0	1	1	$Gly24^{MS}$ $Gly24^{MM}$
Thr254	0	—	2	$Glu290^{SS}$ $Gln250^{MM}$
Asn286	0	1	2	$Glu287^{SS}$ $Ala282^{MM}$ $Glu290^{MM}$
Leu (substrate)	0	2	2	$Gly26^{MM}$ $Tyr108^{SM}$ $Phe253^{MM}$ $Ser256^{MS}$
**Na2:**				
Gly20	0	—	1	$Leu16^{MM}$
Val23	3.17	—	1	$Ala19^{MM}$
Ala351	0	—	1	$Leu347^{MM}$
Thr354	0	1	2	$Phe350^{SM}$ $Phe350^{MM}$ H_2O^{HM}
Ser355	0	2	2	$Asn21^{SM}$ $Gly352^{MM}$ $Ala358^{MM}$ $Ile359^{MM}$
**Aspartate transporter GltPh 3KBC**				
**Na1:**				
Gly306	0	—	1	$Thr302^{MM}$
Asn310	1.2	1	—	$Thr92^{SS}$
Asn401	0	1	2	$Gly313^{MS}$ $Arg397^{MM}$ $Asp500^{SM}$
Asp405	3.0	3	1	$Val402^{MM}$ $Gly408^{MM}$ $Thr409^{MM}$ $Thr409^{SM}$
**Na2:**				
Thr308	47.5	—	2	$Leu305^{SM}$ $Leu305^{MM}$
Ser349	0.3	—	3	$Ala345^{MM}$ $Ala345^{SM}$ $Val346^{SM}$
Ile350	16.8	—	1	$Val346^{MM}$
Thr352	1.3	—	2	$Ala348^{MM}$ $Ala348^{SM}$
**Dopamine transporter DAT 4XP1**				
**Na1:**				
Ala44	0	—	1	$Val40^{MM}$
Asn49	0.1	1	2	$Asp46^{MS}$ $Ser356^{SS}$ $Asp46^{MM}$
Ser320	1	—	1	$Gln316^{MM}$
Asn352	0.7	1	2	$Thr348^{MM}$ $Ser349^{MM}$ $Ser356^{MM}$
**Na2:**				
Gly42	0.5	—	1	$Leu38^{MM}$
Val45	0.9	—	1	$Ile41^{MM}$
Leu417	0.5	—	2	$Met413^{MM}$ $Leu414^{MM}$
Asp420	0	3	—	$Thr268^{SS}$ $Gly424^{MM}$ H_2O^{HM}

Ser421	0	1	2	Phe43^{SM} Asp46^{SS} Gly425^{MM}
**Glutamate transporter GltTk 5E9S**				
**Na1:**				
Gly309	0	1	1	Asn313^{MM} Thr305^{MM}
Asn313	0	—	3	Asn313^{SM} (intraresidue HB) Gly309^{MM} Asp409^{SS}
Asn405	0	2	1	Gly316^{MS} Thr317^{MS} Thr317^{SS}
Asp409	1.8	3	1	Asp409^{SS} Thr413^{MM} Thr413^{SM} Val406^{MM}
**Na2:**				
Thr311	0.3	—	1	Leu308^{MM}
Ser352	0.1	—	4	Ala348^{MM} Ala348^{SM} Val349^{SM} Met314^{SM}
Ile353	7.1	—	1	Val349^{MM}
Thr355	0	—	2	Ala351^{MM} Ala356^{SM}
**Na3:**				
Tyr91	1.2	—	2	Thr305^{SM} Ile87^{MM}
Thr94	1.2	2	3	Tyr90^{SM} Asp315^{SS} Tyr90^{MM} Ala98^{MM} Gln244^{SM}
Ser95	2.6	2	—	Ala98^{MM} Val99^{MM}
Asn313	0	—	3	Gly309MM Asn313^{SM} (intraresidue HB) Asp409SS
Asp315	0	4	—	Leu319^{MM} Thr94^{SS} Gln244^{SS} Tyr249^{SS}
**Serotonin transporter SERT 5I71**				
**Na1:**				
Ala96	0.9	—	1	Val92^{MM}
Asn101	1	—	3	Asn368^{SM} Ser372^{SS} Asp98^{MS}
Ser336	4.7	1	1	Gln332^{MM} Asn368^{SS}
Asn368	6.7	3	2	Asn101^{SM} Ser372^{MM} Ser372^{SM} Ser336^{SS} Thr364^{MM}
**Na2:**				
Gly94	2.4	—	1	Leu90^{MM}
Val97	3.8	—	1	Ile93^{MM}
Leu434	0.7	—	2	Met430^{MM} Leu431^{MM}
Asp437	3	2	1	Thr284^{SS} Asp437^{MS} (intraresidue HB) Ala441^{MM}
Ser438	0	1	1	Tyr95^{SM} Gly442^{MM}
**Glutamate transporter GLAST1/EAAT 5LM4**				
Thr376	7	—	—	—
Ser417	0	—	2	Met379^{SM} Ala413^{MM}
Ala420	1.9	—	1	Ala416^{MM}
Ile418	34.9	—	1	Thr414^{MM}
**Glutamate receptor Kainate GluR5 (K1) 3C32**				
Glu96	0	2	1	Thr92^{MM} Phe101^{MS} Lys103^{SS}
Ile99	0	—	1	Arg95^{MM}
Asp100	0	5	1	Gly223^{MS} 3H_2O^{HS} 2Thr220^{MM}

**Glutamate receptor Kainate GluR6 (K2) 3G3F**				
Glu97	0	3	1	$Thr93^{MM}$ $Lys104^{SS}$ $Arg228^{SS}$ H_2O^{HS}
Ile100	0	—	1	$Arg96^{MM}$
Asp101	0	5	1	$Gly224^{MS}$ $Arg228^{SS}$ $2H_2O^{HS}$ $Thr221^{MM}$ $Thr221^{MM}$
**Glutamate receptor Kainate K5 (KA2) 3OM1**				
Phe162	0	—	1	$Leu158^{MM}$
Ser165	5.3	1	—	$Glu167^{MS}$
Glu167	15.6	1	1	$Ser165^{MS}$ H_2O^{HS}
**Glutamate receptor Kainate GluR7 (K3) 3S9E (chain B)**				
**Na1:**				
Ser24	24.9	1	—	H_2O^{HM}
Arg26	54.9	—	2	$Asp32^{SS}$ H_2O^{SH}
**Na2:**				
Asn5	80.7	2	—	$2H_2O^{HS}$
Ser52	31.3	1	1	$Ser7^{SS}$ $Leu8^{MM}$
**GPCR receptor Protease-activated PAR1 3VW7**				
Ser189	2.3	1	3	$Asn185^{MM}$ $Asn185^{SM}$ $Asn185^{SS}$ $Met193^{MM}$
Asp148	0.4	2	1	$Leu144^{MM}$ $Val152^{MM}$ $Asn185^{SS}$
Asp367	1.7	3	1	$Ser363^{MM}$ $Ile370^{MM}$ $Tyr371^{MM}$ H_2O^{HS}
**GPCR receptor Adrenergic b1AR 4BVN**				
**Na1:**				
Cys198	20	—	1	$Asp195^{MM}$
Cys192	6.1	1	1	$Asp195^{MM}$ $Gln188^{MM}$
Asp195	42.4	2	2	$Lys191^{MM}$ $Cys192^{MM}$ $Cys198^{MM}$ $Gly197^{MS}$
**Na2:**				
Asp87	0	5	1	$Leu83^{MM}$ $Gly91^{MM}$ $Ser336^{SS}$ $3H_2O^{HS}$
Ser128	0.2	1	2	$Cys124^{MM}$ $Cys124^{SM}$ $Leu132^{MM}$
**GPCR receptor Adenosine A2AAR 4EIY**				
Asp52	0	4	—	$Ser281^{SS}$ $3H_2O^{HS}$
Ser91	0	—	1	$Leu87^{SM}$
**GPCR receptor Adrenergic b2AR 4LDE**				
Asn1103	5.8	1	2	$Glu1188^{MS}$ H_2O^{MH} $Glu1107^{MM}$
Glu1187	28	1	1	$Asn1183^{MM}$ $Thr1189^{SS}$
Cys1184	2.9	—	1	$Glu1180^{MM}$
Cys1190	8.4	—	—	—
**GPCR receptor Opioid d-OR 4N6H**				
Asp95	0	6	1	$Ser311^{SS}$ $Asn314^{SS}$ $2H_2O^{HS}$ $Thr99^{MM}$ $Thr99^{SM}$ $Leu91^{MM}$
Ser135	0	1	2	$Asn131^{MM}$ $Asn131^{SM}$ $Leu139^{MM}$
Asn131	1.1	2	3	$Ile127^{MM}$ $Ser135^{MM}$ $Ser135^{SM}$ $2H_2O^{SH}$

GPCR receptor Protease-activated PAR2 5NDD				
Asp121	0.4	2	1	Leu117^{MM} Ser124^{MM} Val125^{MM}
Asp340	0	3	1	Asn336^{MM} Val343^{MM} Tyr344^{MM} H_2O^{HS}
Ser162	0	1	2	Asn158^{MM} Asn158^{SM} Leu166^{MM}
Asn336	0.6	2	2	Ser333^{MM} Ser333^{SM} Ile339^{MM} Asp340^{MM}
